# Improving movement behavior in office workers: effects of two multi-level cluster-RCT interventions on mental health

**DOI:** 10.1186/s12889-024-17647-2

**Published:** 2024-01-09

**Authors:** Lisa-Marie Larisch, Victoria Blom, Maria Hagströmer, Maria Ekblom, Örjan Ekblom, Jonna Nilsson, Lena V. Kallings

**Affiliations:** 1 Department of Physical Activity and Health, The Swedish School of Sport and Health Sciences, Stockholm, Sweden; 2 Division of Insurance Medicine, Department of Clinical Neuroscience, Karolinska Institutet, Stockholm, Sweden; 3 Division of Physiotherapy, Department of Neurobiology, Care Sciences and Society, Karolinska Institutet, Stockholm, Sweden; 4 Academic Primary Health Care Centre, Region Stockholm, Stockholm, Sweden; 5 Department of Health Promoting Science, Sophiahemmet University, Stockholm, Sweden; 6 Department of Neuroscience, Karolinska Institutet, Stockholm, Sweden; 7 Division of Family Medicine and Preventive Medicine, Department of Public Health and Caring Sciences, Uppsala University, Uppsala, Sweden

**Keywords:** Workplace health promotion, Office workers, Physical activity, Sedentary behavior, Behavior change, Mental health, Mental wellbeing

## Abstract

**Background:**

We have previously reported on the design and efficacy of two cluster-randomized multi-level workplace interventions, attempting to decrease sedentary behavior (SED) or increase moderate to vigorous physical activity (MVPA) among office workers to improve mental health outcomes. The aim of this study was to investigate intervention effects on mental health outcomes, i.e., mental wellbeing, depression or anxiety symptoms, and stress immediately after the 6-month intervention period.

**Methods:**

Teams of 263 office workers were cluster-randomized to one of two interventions or a waitlist control group. The PA intervention (iPA) focused on increasing MVPA and the SED intervention (iSED) on reducing SED. Both multi-level interventions targeted individual office workers and their social, physical, and organizational work environment, incorporating counseling based on cognitive behavioral therapy and motivational interviewing. Mental health outcomes were assessed using validated questionnaires before and immediately after the intervention. Intervention effects were analyzed using linear mixed effects models.

**Results:**

Participants were mostly female and highly educated, with a mean age of 42 years and had favorable levels of mental health at baseline. Mental wellbeing improved for the iSED group (β = 8, 95% CI 1 to 15, *p* = 0.030) but not for the iPA group (β = 6, 95% CI -1 to 12, *p* = 0.072) compared to the control group. No effects were found for depression or anxiety symptoms or stress.

**Conclusions:**

The multi-level interventions improved mental wellbeing among this population of office workers, reaching statistical significance in the iSED group. The size of the effect can be regarded meaningful, considering favorable mental health and high PA level at baseline. Thus, workplace interventions that provide support on multiple levels appear to have potential for improving mental wellbeing, but not reducing ill-health variables, among healthy office workers. More research is needed to understand the mechanisms through which such improvements can be achieved and to identify the most effective intervention components.

**Trial registration:**

ISRCTN92968402 (27 February 2018).

**Supplementary Information:**

The online version contains supplementary material available at 10.1186/s12889-024-17647-2.

## Introduction

Impaired mental health presents a significant global health challenge, placing a substantial burden on affected individuals and resulting in notable costs through sickness absences and reduced productivity [1]. There is an urgent need to mitigate the burden of impaired mental health, and workplaces can play an important role in mental health promotion since many working-age adults can be reached via the workplace [1]. Employers in some Western countries are legally obliged to ensure working conditions that prevent not only physical but also mental health impairment [2].

Physical activity (PA) and sedentary behavior (SED) are important determinants of mental health; numerous studies confirmed positive effects of PA and negative effects of extensive SED on various mental health outcomes, such as mental wellbeing, depression or anxiety symptoms, and stress [3–7]. Considering this evidence, office workers may be at an increased risk of poor mental health since they spend up to 82% of work time sedentary [8–10]. In addition, sedentariness at work is associated with sedentariness during non-work time [8]. Thus, it is important to investigate whether office workers’ mental health can be improved by supporting them in reducing SED by interrupting long periods of sitting or by replacing sitting with standing or light-intensity physical activity (LIPA), or by supporting them in increasing time spent in moderate to vigorous physical activity (MVPA), for example by engaging in structured exercise or active commuting. However, only few studies have investigated whether workplace-delivered interventions focusing on SED or PA can also improve mental health outcomes [11–14] and the World Health Organization (WHO) recommends them only conditionally as workplace mental health promotion strategies due to very low certainty of evidence [1].

Workplace-delivered interventions among office workers can lead to meaningful reductions in SED of up to 100 min per 8 h workday [15, 16]. The evidence regarding effectiveness of workplace interventions among office workers focusing on increasing MVPA is inconsistent [17, 18]. The available evidence indicates that interventions which focus on such movement behaviors may reduce symptoms of depression or anxiety, relieve stress [11], and improve mental wellbeing [12] and mental health-related quality of life [14]. However, systematic reviews of previous interventions point to a lack of high-quality randomized controlled trials (RCTs) with adequate sample sizes and control conditions, which might explain the inconsistency of results across studies [11–13]. In addition, there are considerable challenges with previous workplace interventions among office workers that aimed at increasing MVPA or reducing SED. For example, time spent in these different movement behaviors has predominantly been measured with self-reports that are less precise and reliable than accelerometers due to, for example, a social desirability or recall bias [19]. Furthermore, previous interventions usually do not take individual preferences of participants into account, offering one standardized program for all participants [18, 20, 21]. Most interventions that focus on reducing SED make changes to the physical work environment, for example by providing sit-stand-desks, only sometimes in combination with behavioral support such as self-monitoring or goal setting [16, 20, 22]. It might be that previous studies have not achieved desired effects because they do not take individual needs and preferences regarding the choice of activity into account and do not provide sufficient behavioral support for changing behavior. Engaging in activities that people enjoy and find personally meaningful increases the likelihood of establishing and sustaining new behaviors [23], and might be especially important for improving mental health outcomes via changes in movement behavior [24]. Previous literature suggests that factors such as enjoyment, mastery of skills or goals, choice, social interaction, and a sense of belonging likely influence the relationship between PA and mental health [25].

Furthermore, previous literature hypothesized that multi-level interventions including several components that provide support not only on the individual but also via the physical and social work environment and organization might be more efficacious compared to those that only address one of these levels of influences [22, 26]. This is in line with widely established ecological models of health behavior which propose that movement behavior is determined by factors at these different levels [27]. Very few multi-level RCT interventions among office workers investigated whether interventions targeting SED or PA can lead to improved mental health outcomes [28–30]. The results of these studies suggest that multi-level interventions can improve sitting time [28–30] and LIPA [29], daily anxiety, occupational fatigue, job performance, work engagement [28], stress, wellbeing and vigor [30].

Several biochemical mechanisms have been suggested for explaining the mental health effects of increased PA on certain mental health outcomes, such as changes in neuroplasticity, neuroendocrine responses, or reductions in inflammatory processes [31]. In addition, psychosocial mechanisms have been suggested, such as increased self-efficacy, self-esteem or social support [31]. However, it is yet unknown in how far these biochemical or psychosocial mechanisms are depending on PA intensity [32]. Therefore, more research is needed to establish whether interventions among office workers should focus on reducing SED by increasing LIPA, or whether increases in MVPA are necessary for improving mental health outcomes.

We designed a 3-arm multi-level cluster RCT among office workers that aimed to increase MVPA (iPA intervention) or reduce SED (iSED intervention) to improve mental health outcomes among office workers [33].

PA and SED were considered as proximal outcomes because the primary aim of the interventions was to support changes in movement behavior. Thus, PA and SED were expected to be directly impacted by the interventions. Mental health outcomes were instead considered distal outcomes since we originally hypothesized to impact mental health via changes in PA and SED. However, previous analyses based on the same RCT showed that participants already had high levels of accelerometer-measured PA (approximately 1.5 h of MVPA per day) but also high levels of SED (approximately 13 h) at baseline, despite efforts to enroll less active office workers [34]. The interventions had no average effects on accelerometer-measured MVPA, SED [35], 24-h movement behavior [34], movement behavior during work or non-work time [34], cardiorespiratory fitness [34] or cognitive functions [36]. However, the interventions increased autonomous and controlled motivation among iPA participants [37]. Self-efficacy in relation to regulating own movement behavior also improved among participants who attended at least three of the five counseling sessions [37].

Thus, we hypothesize that the interventions could elicit changes in mental health outcomes via mechanisms that are not directly related to the intended changes in movement behavior, but via activation of psychosocial mechanisms such as increased self-efficacy or social support [31]. This hypothesis is further supported by the fact that the interventions included counseling sessions based on cognitive behavioral therapy (CBT) and motivational interviewing (MI) to provide individual-level support for increasing PA or reducing SED [38]. CBT is an evidence based method for changing health behaviors and improving mental health outcomes [39, 40]. The counseling sessions aimed at helping individuals understand how thoughts and emotions impact their movement behavior and at increasing their self-efficacy for finding and sustaining new helpful behaviors that are in line with their personal preferences and values. MI is a client-centered, goal-oriented counseling approach that seeks to elicit and strengthen a person's intrinsic motivation for positive behavior change by exploring and resolving ambivalence [41]. As the interventions might have impacted on mental health also directly and not only via changes in movement behavior,To our knowledge, only a few studies with small sample sizes have used CBT to support movement behavior change; existing studies among healthy older adults showed that CBT improves exercise behavior and increases adherence to predefined exercise programs [42, 43]. To our knowledge, no previous interventions have included CBT and MI for the specific aim of supporting movement behavior change to improve mental health outcomes among office workers.

The aim of this study was to investigate the effect of multi-level workplace interventions focusing on increasing MVPA or reducing SED on mental wellbeing, depression or anxiety symptoms, and stress.

## Methods

### Study design

This study aimed to investigate intervention effects on distal outcomes of a 6-month, three-armed cluster RCT among 263 office workers from two Swedish companies in the retail and finance sector. Because outcomes were considered distal and because the RCT did not reach the enrollment target of *N* = 330 participants [33], analyses should be seen as exploratory rather than confirmatory. A detailed description of the RCT, including information about sample size calculation and randomization procedures, can be found in the published study protocol [33]. This study was performed according to the Helsinki Declaration. Ethical approval was granted by the Stockholm Regional Ethical Review Board (2017/2409–31/1). All participants provided written informed consent before the first data collection. The trial was prospectively registered as ISRCTN92968402 on 27 February 2018, and recruitment started on 15 March 2018 (https://doi.org/10.1186/ISRCTN92968402, accessed on 13 April 2021). The trial was completed in 2020 before the start of the COVID-19 pandemic. The study was conducted in accordance with the CONSORT guidelines for cluster RCTs.

### Participants

All employees of the two participating companies (*N* = 2033) received oral and written information about the study and an invitation to participate via email [33]. The inclusion criteria were ages 18–70 and the ability to stand and move. This RCT aimed at less active office workers who might profit most from the interventions. Therefore, individuals who were highly physically active, defined as spending more than 30 min/day in MVPA in bouts of at least 10 min, on each day of the 7-day baseline accelerometer measurement, were excluded (*N* = 10). Another exclusion criterion was not working for the full duration of the first study year due to for example retirement or parental leave (*N* = 0).

### Intervention design

**Fig. 1:**
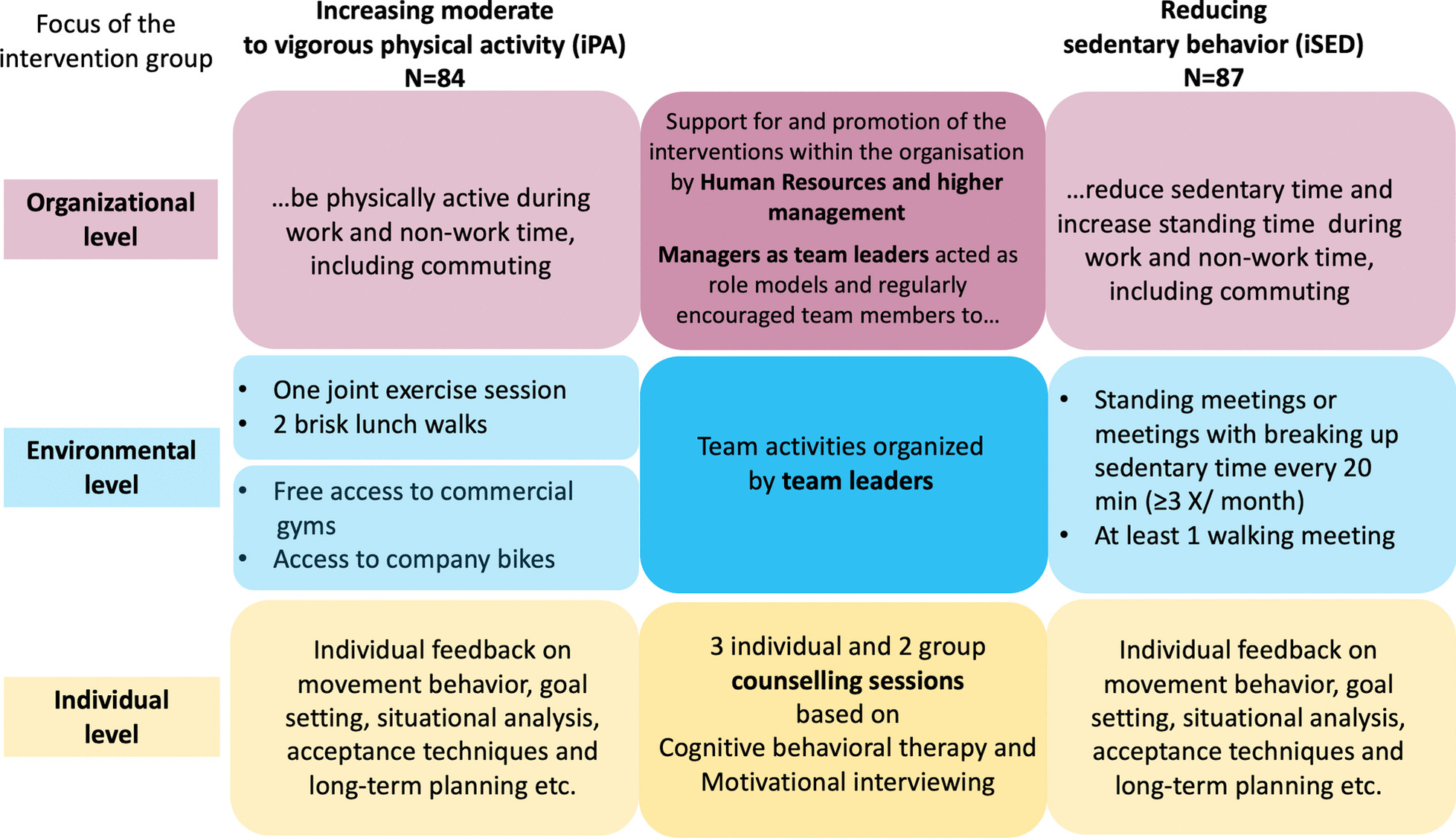
Overview of the 6-month multi-level interventions. Columns describe The intervention activities in the iPA group, Activities that both groups received and Activities in the iSED group [45]

The companies’ human resource (HR) staff in collaboration with one of the researchers grouped participants into 22 cluster teams based on the criteria of having a team or line manager, regular group meetings, and limited regular meetings with other teams. Cluster teams were randomized to one of two intervention groups or a wait-list control group that received one of the interventions after the initial 6-month intervention period [44]. One intervention group focused on increasing MVPA (iPA) and the other on reducing SED (iSED) by interrupting prolonged bouts of sitting and replacing SED with LIPA. Based on ecological models of health behavior [27], the interventions included multiple components to provide support on the individual, social, physical, and organizational level, see Fig. 1.

A complete list of the included behavior change techniques according to the taxonomy of behavior change techniques [46] can be found in Supplementary file 1 of the published study protocol [33]. To provide organizational support, HR and higher management staff promoted participation in the interventions within their companies and allowed the use of work time for participation in intervention activities and data collection. We aimed at recruiting managers as team leaders who were asked to provide support by acting as role models, by encouraging respective behaviors, and by promoting continued participation in the interventions. Team leaders were also responsible for implementing team and environmental components: one joint exercise session and lunch walks for iPA teams and standing and walking meetings for iSED teams to increase social support and offer opportunities for more PA and less SED in the work environment. To address the individual level, both groups received three individual and two group counseling sessions, based on CBT and MI. These were led by professional health coaches and delivered face to face. The goal of these sessions was to support participants in understanding the connection between their thoughts, emotions, and movement behavior to increase their self-awareness and self-efficacy for achieving the goal of being more physically active (iPA) or less sedentary (iSED). A detailed manual was used to standardize each session (see Additional file 1). Between sessions, participants were asked to manually track their PA (iPA group) or SED (iSED group) in a logbook and to complete CBT homework assignments. Participants chose activities according to their individual needs and preferences to change movement behavior during work, non-work time and commuting. Participants did not receive any compensation for their participation. However, they were allowed to partake in intervention activities and data collection during working hours at the workplace. To mitigate the influence of seasons, the start of the 6-month intervention period for the different teams was spread across seasons.

### Data collection

At baseline and after the 6-month intervention period, participants completed a web-based survey including validated mental health questionnaires and questions about demographic characteristics. Data from the 12 months follow-up was not used due to a very low participation rate.

#### Measurement of mental health outcomes

The 14-item Hospital Anxiety and Depression Scale (HADS) is commonly used to assess depression and anxiety symptoms among diverse populations to identify potential early signs of distress but also as a screening tool [47]. Symptoms of depression or anxiety were included since these mental health disorders are the most common ones. Answers from the 4-point Likert scale are summed. Scores of 0–7 signify no presence of clinical symptoms, 8–10 borderline abnormal symptoms, and 11–21 abnormal symptoms [47].

The 5-item World Health Organization-Five Well-Being Index (WHO-5) was used to assess mental wellbeing [48]. It is designed to measure positive aspects of mental health, such as emotional well-being, vitality, and satisfaction with life, rather than symptoms of mental disorder [48]. Answers from the 6-point Likert scale are summed and multiplied by four [49, 50]. Sum scores range from 0 to 100, with higher values indicating better wellbeing. Summed scores of > 50 indicate good mental wellbeing, ≤ 50 indicate low mental wellbeing although not necessarily depression, and scores of ≤ 28 indicate likely depression, indicating the need for further clinical assessment. An improvement of 10 points is considered clinically relevant [49, 50]. For the RCT, one item was modified to explore differences in wellbeing related to private vs. work life. Instead of *…my daily life has been filled with things that interest me*, two statements were created: my *daily private life* and *my daily work life*. For this study, a mean of the two answer options was calculated and used for the sum score.

Stress is an important work-related health risk factor and was assessed using the single-item stress question, asking how often a person experienced stress during the past week on a 5-point Likert scale [51]. Note that we treated the stress variable as an ordinal approximation of a continuous variable [52]. The three mental health scales have high validity and reliability among healthy adult populations [49, 53, 54].

### Statistical analysis

#### Descriptive statistics

Mean and standard deviation (SD) were calculated to describe age and years of education, and proportions were calculated to describe gender as key demographic characteristics. Median and interquartile range (IQR) were calculated for baseline and follow-up values for depression and anxiety symptoms due to their skewed distributions. Mean and SD were calculated for mental wellbeing and stress. For descriptive purposes, we categorized values for depression and anxiety symptoms and mental wellbeing using the above-described validated cutoffs. Baseline differences between groups for age, gender and education were assessed using Tukey’s pairwise comparison.

#### Missing data analysis

Participants with missing data for mental health outcomes at baseline or follow-up were compared to those with complete data. For each analytical sample, we compared baseline age, gender, years of education, mental health outcomes and group affiliation (iPA, iSED or control) between participants with complete vs. missing data using Student’s t-test and chi-square test.

#### Hierarchical linear mixed effects models

Linear mixed effects model analysis was performed to account for the clustered and hierarchical structure of the data, i.e., individuals were nested in clusters. Randomization into three groups (iPA, iSED and control) was significantly associated with missingness of data on mental health outcomes at baseline or follow-up. Thus, data were not missing at random, and maximum likelihood estimation including participants with missing data was contraindicated. Therefore, complete case analysis was performed using five different analytical samples, one for each mental health outcome. *N* = 158 participants had complete data for mental wellbeing, *N* = 140 for depression symptoms, *N* = 140 for anxiety symptoms, and *N* = 163 for stress.

To investigate intervention effects, one linear mixed effects model was fitted for each mental health outcome. For the main analyses, we explored intervention-specific effects for the iPA and iSED intervention in comparison to the control group.

#### Random effects

The final two-level hierarchical models included a random intercept for clusters (higher level 1) and individuals (lower level 2), which were nested within clusters. Including these random effects accounted for the variability within and between individuals and clusters. Data points were not sufficient for modeling random slopes for clusters and individuals. To test the significance of the random effects, analysis of variance (ANOVA) was used to compare models with and without the random effects. We used the Akaike Information Criterion (AIC) to evaluate whether the model with or without random intercepts would best fit the data.

#### Fixed effects

We estimated the fixed effects of intervention group (3 levels: iPA, iSED vs. control), time (2 levels: baseline vs. follow-up), and interaction between intervention group and time (3 × 2).

Tukey pairwise comparison was used to analyze baseline differences in mental health outcomes between groups and to compare intervention effects between the iPA and iSED groups.

We applied an unstructured covariance structure to optimize the model fit. To evaluate model fit, the normal distribution of residuals was visually inspected post hoc to confirm that the normality assumptions for linear regression were fulfilled. In addition, fitted values were plotted against residuals to inspect linear and equal error variance and outliers. A *p*-value of ≤ 0.05 was set as the level of statistical significance. All statistical analyses were conducted in R (Version 3.6.3) [55]. Linear mixed effects models were fitted using the lme4 package [56].

## Results

### Baseline characteristics and missing data analysis

**Fig. 2:**
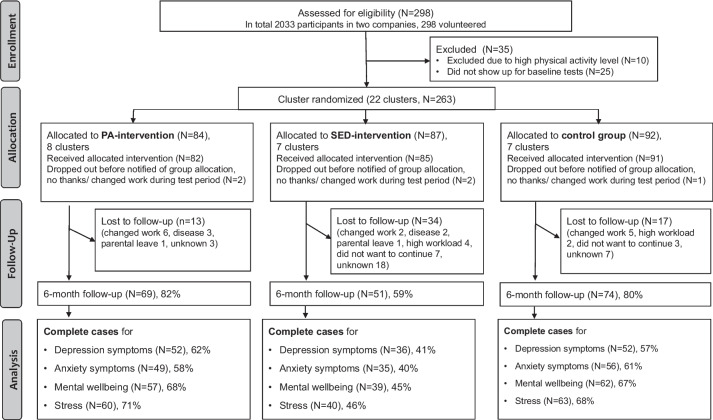
Flowchart for enrollment, participation, and analysis

Figure 2 presents a flowchart for enrollment, participation, and analysis. One hundred and fifty-eight participants had complete baseline and follow-up data for mental wellbeing (60% of all RCT participants), 140 for depression (53% of all RCT participants), 140 for anxiety (53% of all RCT participants), and 163 for stress (62% of all RCT participants). The proportion of those with missing data was larger in the iSED group than in the iPA and control group in all analytical samples.

Table 1 presents the participants’ baseline demographic characteristics per analytic sample. Participants were on average 42 years old (SD 8), with control group participants being significantly older. Participants were, on average, highly educated, and approximately 75% were female.

**Table 1:** Participants’ baseline demographic characteristics per analytic sample

	*All participants*	*iPA*	*iSED*	*Control group*
***Depression symptoms***	***N* = 140**	***N* = 52**	***N* = 36**	***N* = 52**
*Age, mean (SD) years*	42 (8)	40 (8)	40 (8)	45 (8)^a^
*Male, N (%)*	32 (29)	11 (21)	9 (25)	12 (23)
*Education, mean (SD) years*	15 (2)	15 (2)	15 (2)	15 (2)
***Anxiety symptoms***	***N* = 140**	***N* = 49**	***N* = 35**	***N* = 56**
Age, mean (SD) years	42 (8)	41 (9)	40 (8)	44 (7)^a^
Male, N (%)	35 (25)	11 (22)	9 (26)	15 (27)
Education, mean (SD) years	15 (2)	15 (2)	15 (2)	15 (2)
***Mental wellbeing***	***N* = 158**	***N* = 57**	***N* = 39**	***N* = 62**
*Age, mean (SD) years*	42 (8)	41 (9)	41 (8)	45 (8)^a^
*Male, N (%)*	40 (25)	11 (19)	12 (31)	17 (27)
*Education, mean (SD) years*	15 (2)	15 (2)	15 (2)	15 (2)
***Stress***	***N* = 163**	***N* = 60**	***N* = 40**	***N* = 63**
*Age, mean (SD) years*	42 (8)	41 (9)	40 (8)	45 (7)^a^
*Male, N (%)*	40 (25)	11 (18)	12 (30)	17 (27)
*Education, mean (SD) years*	15 (2)	15 (2)	15 (2)	15 (2)

*iPA* Physical activity intervention, *iSED* Sedentary behavior intervention, *SD* Standard deviation

^a^Age at baseline was significantly higher in the control group than in the intervention groups

Table 2 presents baseline and follow-up values for mental health outcomes, specified per allocated group. At baseline, 6% had likely depression, and 28% had poor mental wellbeing according to the WHO-5 scores. According to the HADS, 2% of participants had abnormal depression symptoms, 11% had borderline abnormal depression symptoms; 17% had abnormal anxiety symptoms, and 15% had borderline abnormal anxiety symptoms. 30% of participants experienced stress several times per week or daily, with 6% experiencing stress every day. No baseline differences between groups in mental health outcomes were observed.

**Table 2:** Baseline and follow-up data for outcome variables, specified per allocated group**.** Table includes participants with complete data for the respective outcomes at both baseline and 6-month follow-up

	**All participants**		**Control group**		**iPA**		**iSED**	
	Baseline	Follow-up	Baseline	Follow-up	Baseline	Follow-up	Baseline	Follow-up
**Depression symptoms**	***N* = 140**		***N* = 52**		***N* = 52**		***N* = 36**	
*Median (IQR)*	3.0 [1, 5]	2.0 [1, 5]	3.5 [1, 6]	2.5 [1, 6]	3.0 [2, 5]	2.0 [1, 4]	2.0 [1, 5]	2.0 [1, 3]
*N (%) Normal*	122 (87)	129 (87)	48 (92)	81 (92)	44 (85)	47 (90)	31 (86)	24 (94)
*N (%) Borderline*	15 (11)	10 (7)	4 (8)	6 (7)	7 (14)	5 (10)	5 (14)	1 (3)
*N (%) Abnormal*	3 (2)	1 (1)	0	1 (1)	1 (2)	0	0	1 (3)
**Anxiety symptoms**	***N* = 140**		***N* = 56**		***N* = 49**		***N* = 35**	
*Median (IQR)*	6.0 [4, 9]	6.0 [4, 8]	6.0 [3, 10]	6.0 [4, 8]	6.0 [4, 7]	5.0 [3, 6]	6.0 [5, 910]	6.0 [3, 8]
*N (%) Normal*	95 (68)	101 (72)	36 (64)	35 (63)	37 (76)	42 (86)	22 (63)	24 (69)
*N (%) Borderline*	21 (15)	25 (18)	7 (13)	12 (21)	8 (16)	6 (12)	6 (17)	7 (20)
*N (%) Abnormal*	24 (17)	14 (10)	13 (23)	9 (16)	4 (8)	1 (2)	7 (20)	4 (11)
**Mental Wellbeing**	***N* = 158**		***N* = 62**		***N* = 57**		***N* = 39**	
*Mean (SD)*	58.05 (16.92)	60.89 (16.81)	56.74 (17.56)	55.55 (18.04)	59.86 (16.38)	64.49 (15.51)	57.49 (16.84)	64.10 (14.65)
*N (%) Good mental wellbeing*	104 (66)	118 (75)	38 (61)	40 (65)	39 (68)	47 (62.5)	27 (69)	31 (79.5)
*N (%) Poor mental wellbeing*	44 (28)	30 (19)	19 (31)	14 (23)	15 (26)	8 (14)	10 (26)	8 (20.5)
*N (%) Likely depression*	10 (6)	10 (6.3)	5 (8)	8 (13)	3 (5)	2 (3.5)	2 (5)	0
**Stress**	***N* = 163**		***N* = 63**		***N* = 60**		***N* = 40**	
Mean (SD)	2.75 (1.14)	2.66 (1.17)	2.65 (1.18)	2.71 (1.26)	2.87 (1.14)	2.60 (1.09)	2.75 (1.10)	2.65 (1.14)
Less than a few times per month N (%)	25 (15)	30 (18)	10 (16)	13 (21)	9 (15)	11 (18)	6 (15)	6 (15)
A few times per month, N (%)	47 (29)	49 (30)	24 (38)	18 (29)	12 (20)	17 (28)	11 (27.5)	14 (35)
Once per week N (%)	43 (26)	40 (25)	11 (17.5)	10 (16)	21 (35)	19 (32)	11 (27.5)	11 (27.5)
Several times per week, N (%)	39 (24)	35 (22)	14 (22)	18 (29)	14 (23)	11 (18)	11 (27.5)	6 (15)
Every day, N (%)	9 (6)	9 (6)	4 (6)	4 (6)	4 (7)	2 (3)	1 (3)	3 (8)

*iPA* Physical activity intervention, *iSED* Sedentary behavior intervention, *SD* Standard deviation, *IQR* Inter quartile range

Higher scores for depression, anxiety, and stress indicate worse symptoms. Higher scores for mental wellbeing indicate better mental wellbeing

Median and IQR reported for depression and anxiety due to non-normal distributions

Validated cut-offs used for classifying mental wellbeing and depression or anxiety symptom. Stress presented according to the five answer options

### Effects on mental health outcomes

At the 6-month follow-up, mental wellbeing had improved for the iSED group (β = 7.8, 95% CI 0.7 to 14.8, *p* = 0.030) compared to the control group but not for the iPA group (β = 5.8, 95% CI -0.5 to 12.2, *p* = 0.072). The difference between intervention groups was not statistically significant (β = -1.9, 95% CI -10.5 to 6.5., *p* = 0.848).

No intervention effects were found for depression (β = -0.4, 95% CI -1.3 to 0.6, *p* = 0.470) or anxiety (β = -0.6, 95% CI -1.5 to 0.2, *p* = 0.125) symptoms or stress (β = -0.3, 95% CI -0.6 to 0.1, *p* = 0.131) (see Table 3). The random effects were always significant and improved the model fit based on the AIC.

**Table 3:** Beta coefficients for differences in change from baseline to 6-month follow-up for mental health outcomes *between* groups

***Differences in change between groups***	**iPA vs control** ß (95% CI)* P value*	**iSED vs control** ß (95% CI)* P value*
**Depression**	-0.4 (-1.5 to -0.7)* p* = 0.488	-0.3 (-1.5 to -0.9)* p* = 0.606
**Anxiety**	-0.9 (-1.8 to 0.1)* p* = 0.070	-0.3 (-1.4 to 0.7)* p* = 0.522
**Mental wellbeing**	5.8 (-0.5 to 12.2)* p* = 0.072	**7.8 (0.7 to 14.8)* p* = 0.030**
**Stress**	-0.3 (-0.7 to 0.1)* p* = 0.092	-0.2 (-0.6 to 0.3)* p* = 0.457

*iPA* Physical activity intervention, *iSED* Sedentary behavior intervention, *CI* Confidence interval

Beta coefficients for time*group interactions with 95% confidence intervals from the linear mixed models adjusted for age, gender, and education

Higher scores for depression, anxiety and stress indicate worse symptoms. Higher scores for mental wellbeing indicate better mental wellbeing

Bold values are significant at the *p* < 0.05 level

**Fig. 3:**
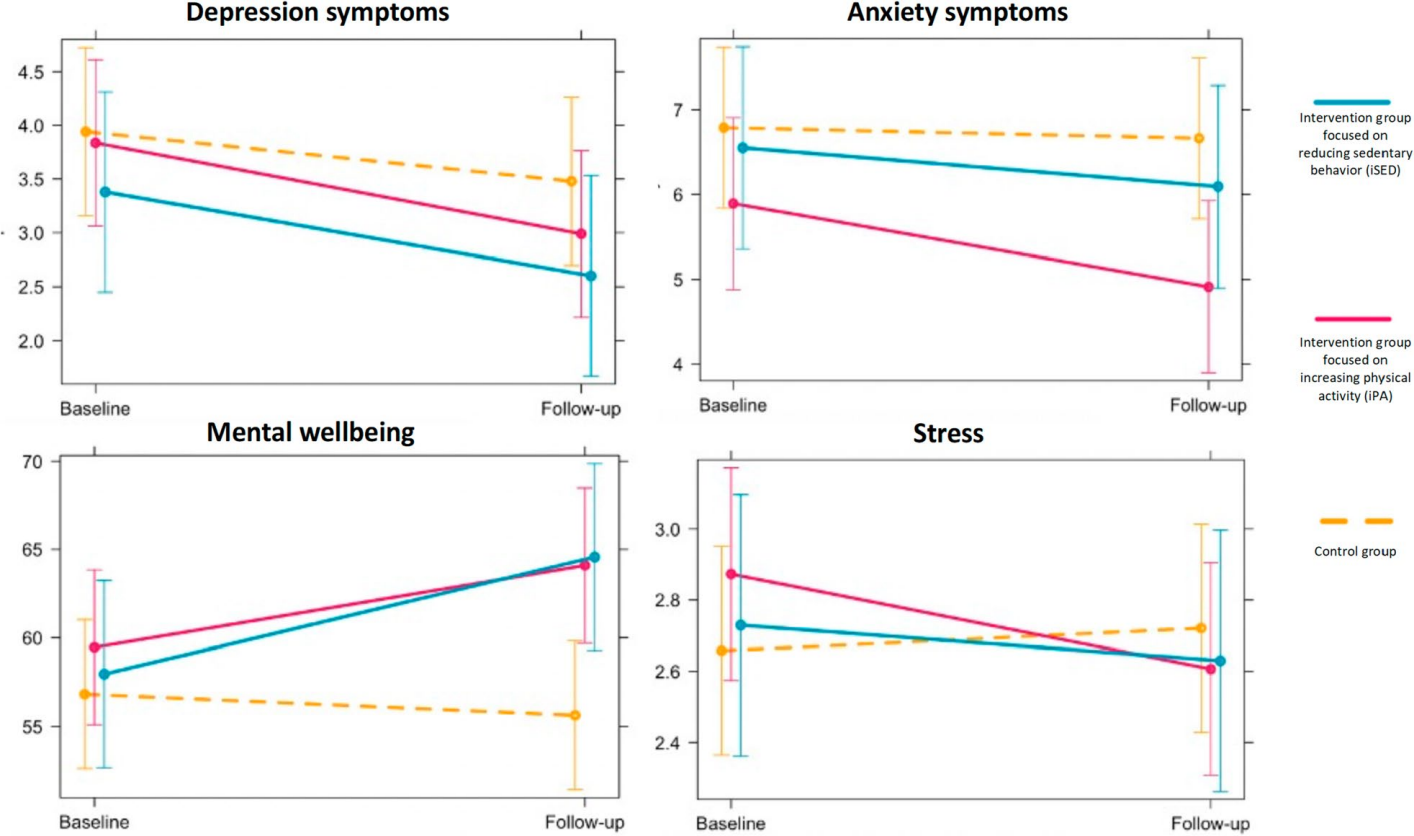
Estimated marginal means plot with 95% confidence intervals for each mental health outcome at baseline and 6-month follow-up. Higher scores for depression, anxiety, and stress indicate worse symptoms. Higher scores for mental wellbeing indicate better mental wellbeing

Figure 3 presents marginal means plots for all mental health outcomes for the separate intervention groups.

## Discussion

The aim of this study was to evaluate the effects on mental health outcomes of two 6-month multi-level interventions among office workers that focused on increasing MVPA or reducing SED. Compared to the control group, the intervention group which focused on reducing SED improved mental wellbeing. Mental wellbeing also increased for the intervention group focusing on increasing MVPA in comparison to the control group but did not reach statistical significance. No intervention effects were found for depression or anxiety symptoms or stress.

### Interpretation of intervention effects on mental wellbeing

Mental wellbeing increased by eight points for the iSED group and by six points for the iPA group. This is below the 10-point improvement that experts suggest as clinically relevant on the individual level [49, 50]. However, the potential public health relevance of intervention effect sizes should be judged in light of the study population’s baseline mental health status, whether interventions are scalable and whether similar effect sizes can be anticipated in the wider target population from which the study population was drawn [57]. Universal interventions, which are designed to benefit high- and low-risk individuals, tend to yield smaller effect sizes compared to interventions specifically targeting high-risk individuals [57]. Smaller health improvements at the group level can still have a significant impact on population health since they apply to a large number of people [57]. Our interventions were delivered by trained health coaches and the companies’ own managers and HR staff. Thus, intervention conditions resembled those that would exist if such interventions were implemented at scale at the workplaces, and similar effect sizes might be found under implementation conditions.

Improvements in mental wellbeing were observed in both intervention groups, but statistically significant intervention effects were found for the iSED group but not for the iPA group with no significant difference between the groups. This might indicate that the iSED intervention was more effective for improving mental wellbeing. In fact, according to the qualitative RCT analysis, iSED participants perceived fewer barriers to reducing their time spent in SED compared to iPA participants who described it as very challenging to integrate more exercise into their everyday lives [45]. In addition, participants spent substantial amounts of time in SED at baseline, suggesting that there was room for improvement, opposite to the already large amounts of time spent in PA at baseline. However, no intervention effects on either movement behavior have been found [34, 35]. Another potential explanation for the difference in intervention effects between intervention groups might be related to retention rates. Drop-out from the iSED group was larger compared to the iPA group, potentially because the iPA intervention was perceived as more attractive since it included free gym cards [45]. Thus, it is possible that only highly motivated iSED participants completed the iSED intervention and provided complete data. This might explain why effects were found for iSED but not iPA participants.

The primary goal of the intervention was to support participants in increasing PA or reducing SED to ultimately improve their mental health. Considering that no interventions effects on PA or SED were found in previous analyses [34, 35], the observed improvements in mental wellbeing might have been caused via other mechanisms. It is possible, that the observed increases in autonomous and controlled motivation, as well as self-efficacy, in relation to regulating own movement behavior led to improved mental wellbeing [37]. In addition, the social and emotional support and attention that participants received through the interventions might have increased feelings of hope or expectations of positive change. Thus, there is a possibility that the interventions might have impacted on mental health also directly and not only via changes in movement behavior. Mental wellbeing might also be more easily influenced by these mechanisms than the other outcomes, i.e., symptoms of depression and anxiety and perceived stress.

Furthermore, the interventions were not placebo-controlled. Thus, we cannot exclude a potential placebo effect, i.e., participants experiencing positive changes in mental wellbeing due to their belief that they are receiving a beneficial treatment, even if the treatment itself had no therapeutic effect [58, 59].

### Interpretation of lack of intervention effect on depression and anxiety symptoms and stress

The population in this study was a generally healthy, non-clinical sample of working adults. The prevalence of depression symptoms was 2% among participants (*N* = 3) compared to pre-pandemic levels of 6% among the general adult Swedish population [60]. Anxiety symptoms were slightly more prevalent among participants (17%) compared to adult Swedish populations (14%) [60]. The proportion of participants experiencing stress at least several times per week or daily at baseline was 30%, which is higher compared to the general Swedish population (15% of women, 10% of men) [61]. Participants had good mental wellbeing at baseline, comparable to populations from other European countries [49, 62–64]. Nonetheless, we found intervention effects on mental wellbeing but not depression or anxiety symptoms or stress. Differences in effects might be explained by the fact that the WHO-5 is designed to assess positive aspects of mental health, such as emotional well-being, vitality, and satisfaction with life, whereas the HADS measures indicators of mental disorder [47]. Changes in mental wellbeing might be easier to achieve than changes in depression and anxiety symptoms among healthy office workers.

In addition, study participants already had higher than average levels of MVPA (approximately 100 min daily on average) [34] at baseline compared to the average Swedish population [65], indicating a potential ceiling effect. Dose‒response studies showed that even small increases in PA can improve depression or anxiety among less active populations, but the effects plateau around one hour of MVPA per day [66], suggesting a curvilinear relationship.

It is also plausible that workplace-delivered movement behavior interventions are efficacious for improving some mental health outcomes, while others require other types of interventions, e.g., addressing workload, organization, or leadership. More research is needed to investigate this.

### Comparison with previous research

The results of this study are partially in line with results from other RCTs among office workers that tested multi-level workplace interventions that were based on ecological models of health behavior. In contrast to our study, most previous studies focused on reducing sitting time and had longer intervention periods [30]. Other RCTs reported improvements in daily sitting time [30], overall SED and leisure PA [67], occupational sitting and standing time [28] and small but significant improvements in mental wellbeing (two points, assessed with the WHO-5) [30], job performance, work engagement, occupational fatigue, sickness presenteeism, daily anxiety, and quality of life [28]. However, one multi-level study did not find effects on total sitting, standing or stepping time and health- or work-related outcomes [68]. Some [30] but not all interventions [67] improved stress but not depression and anxiety symptoms (assessed with the HADS) [30].

Despite previous interventions also adopting multi-level designs, several dissimilarities might explain differences in intervention effects. First, the context, available resources and health culture at the companies differ across companies and are likely to impact the efficacy of such interventions [69–71]. However, such contextual information is usually not provided, and their impact on intervention efficacy not assessed. For example, participants in our study already had sit-stand desks, as is common in Swedish workplaces, whereas the introduction of sit-stand desks was a component of many previous multi-level studies [28, 30, 67, 68]. Available multi-level interventions among office workers also differ by the type and number of included intervention components, which levels of influence they address, the degree of involvement of managers or higher leadership, reach within the company, and intervention duration, all of which might explain differences in efficacy. In fact, manager support has been identified as very important for supporting more PA and less SED in the workplace but was not delivered as planned in our interventions since it was difficult to recruit managers as team leaders for the interventions [45]. Instead, regular employees acted as team leaders. This might have reduced overall intervention efficacy. Some of the previous interventions were more intensive in terms of the number of components included per level of influence. It seems that more intensive interventions regarding the number of included components and frequency of contact with intervention components [28, 30] achieved greater improvements in movement behavior, mental health and work-related outcomes compared to less intensive interventions [68]. Often, these multi-level interventions aim at changing movement behavior as the short-term, proximal outcome. Proximal outcomes are expected to change faster than distal outcomes, which require longer to appear [57]. Thus, a potential explanation for the differences in effects on mental health outcomes between our and other studies might be the differences in the timing of the follow-up measurements.

Another study based on this RCT found that iPA participants with high executive function and perceived high demands but also control in relation to work substantially increased LIPA, and iSED participants with these characteristics showed a tendency of reduced SED compared to the control group [72]. Executive function refers to the higher-level cognitive skills used to control and coordinate other cognitive abilities as well as behavior. This emphasizes the importance of better understanding which individuals benefit from such interventions and how interventions can be tailored to benefit a broader population.

### Implications

The findings from this study have implications primarily for occupational health researchers and employers. Most importantly, our study confirmed that holistic workplace-delivered interventions which provide support for movement behavior change on multiple levels can improve office workers’ mental wellbeing. The CBT and MI techniques were useful in providing individualized support for movement behavior change [45] and their potential should be further evaluated in the context of workplace health promotion.

Since only a few well-designed RCTs have investigated the efficacy of movement behavior interventions on mental health outcomes, more research among diverse groups of office workers is needed to understand their potential for addressing the burden of impaired mental health. Researchers may want to consider the following aspects when designing future similar multi-level interventions. To improve the effectiveness of such interventions, there is a need to identify how (analysis of mediators), for whom (analysis of personal moderators), and under what circumstances (analysis of situational moderators) such interventions can lead to health improvements [73]. Future research may also want to consider including diverse types of office work with varying possibilities for social interactions, degrees of remote work, responsibility, possibility for taking breaks, etc., all of which might impact the efficacy of such interventions. In addition, the office design, e.g., activity-based or large open plan offices vs. smaller unit offices, might affect efficacy.future studies should consider including placebo control groups to better understand the mechanisms by which movement behavior interventions cause improvements in mental health [59]. It is important to consider that universal interventions may produce smaller effect sizes than interventions that focus on at-risk populations, but they might also be less likely to cause stigmatization since participation in such interventions is not determined by mental health status [1].

### Strengths and limitations

This study was based on a large cluster RCT evaluating the mental health effects of interventions that aim at improving movement behavior. The design of the interventions was based on ecological models of health behavior. The interventions were delivered by trained health coaches as well as HR staff and employees acting as team leaders. Therefore, the results might reflect what could be expected if the interventions were implemented at scale.

Several limitations must be considered. First, the study was underpowered for detecting statistically significant changes in mental health outcomes. Two hundred sixty-three office workers were enrolled, but 330 were needed to achieve a power of 80% to detect changes in stress, according to the sample size calculation [33]. Only the stress measure was included in the sample size calculation. Hence, non-significant findings could be attributed to either true absence of intervention effects or the possibility that the effects, if present, were too small to be detected with the given sample size.

Conducting complete case analysis was considered more robust since randomization was related to missingness of data on mental health outcomes, contraindicating maximum likelihood estimation including participants with missing data. However, this further reduced the statistical power.

We found no differences between participants with complete data and those with missing data regarding key demographic characteristics. However, there might have been differences regarding motivation or other unmeasured factors, introducing a potential selection bias. Participants included in the analyses might therefore not have been representative of all included RCT participants.

A selection bias might also have occurred regarding the inclusion of workplace and employees. Participants with good mental health and favorable movement behavior might be more inclined to participate and might find it easier to engage with and enact the intervention components, overestimating the health benefits. However, health benefits for those participants might also be more difficult to achieve given their better mental health and movement behavior, leading to a potential underestimation of intervention effects. In addition, participants in this study were predominantly female, highly educated and 42 years old and potentially not representative of all office workers. Mental health outcomes were assessed via self-report questionnaires and may have been subject to reporting bias.

## Conclusions

The aim of the study was to investigate the effect of multi-level workplace interventions focusing on increasing MVPA or reducing SED on mental health outcomes among office workers. Even in a context of no average change in MVPA or SED following the interventions [34, 35], the iSED intervention led to meaningful improvements in mental wellbeing relative to the control group, with a similar pattern also for the iPA intervention. No effects were found for depression or anxiety symptoms and stress. More research is needed to understand the mechanisms through which improvements in mental wellbeing could be achieved and to identify the most effective intervention components. Nevertheless, it is evident from the present study that workplace interventions providing support on multiple levels have the potential of improving mental wellbeing among healthy office workers.

### Supplementary Information

**Additional file 1.** Manual for supporting change in movement behavior based on cognitive behavioral therapy and motivational interviewing.

**Additional file 2.** CONSORT'2010'checklist'of'information'to'include'when'reporting'a'randomised'trial*

## Data Availability

The datasets used in this study are not publicly available because free access to data was not included in the original approval by the regional ethics board and the informed consent agreements with participants. However, they can be obtained from the corresponding author upon reasonable request.

## Abbreviations

AIC: Aikaike Information Criterion
ANOVA: Analysis of variance
CBT: Cognitive behavioral therapy
HR: Human Resource
LIPA: Light-intensity physical activity
MI: Motivational interviewing
MVPA: Moderate to vigorous physical activity
PA: Physical activity
RCT: Randomized controlled trial
SED: Sedentary behavior
WHO-5: The World Health Organization-Five Well-Being Index
HADS: Hospital Anxiety and Depression Scale
iSED: Sedentary behavior intervention
iPA: Physical activity intervention
