# Supplementary material for: Improving movement behavior in office workers: effects of two multi-level cluster-RCT interventions on mental health
Source: BMC Public Health. 2024 Jan 9;24:127. doi: 10.1186/s12889-024-17647-2 (PMC10985866; doi:10.1186/s12889-024-17647-2)
Supplement: Supplementary file 1 — Additional file 1. Manual for supporting change in movement behavior based on cognitive behavioral therapy and motivational interviewing. [file 12889_2024_17647_MOESM1_ESM.pdf]

MANUAL FOR SUPPORTING CHANGE IN MOVEMENT BEHAVIOR

BASED ON COGNITIVE BEHAVIORAL THERAPY

AND

MOTIVATIONAL INTERVIEWING

Timing of the counselling sessions

Week 0 Baseline measurements

Week 1-2 Session 1 Individual session (60min)

Week 3-4 Session 2 Individual session(45min)

Week 7-10 Session 3 Group session (90min)

Week 13 Session 4 Individual session (45min)

Week 24 Session 5 Group session (90min)

Week 24 Follow-up measurements

Overview

Session 1 Education about physical activity and health, map the current situation and goal formulation linked to values (individual)

Session 2 Introduce situation analysis (individual)

Session 3 Experience-based practice in acceptance (group)

Session 4 Exploring barriers and alternative behaviors (individual)

Session 5 Summary and maintenance of behavior change (group)

Additional material

Please note that these additional files are available upon request (email victoria.blom@gih.se)

- Additional file 1. Physical activity and sedentary behavior diary
- Additional file 2. ACT matrix. Participants tie goals to internal rewards and values, and identify individual resources and barriers for behavior changes
- Additional file 3. VAS-scale
- Additional file 4. Situation analysis
- Additional file 5. Alternative behaviors
- Additional file 6. Summary and maintenance plan for sustaining behavior change
- Additional file 7. Checklist

Session 1 - Individual session

1. **Transferring knowledge about physical activity/sedentary activity and health as well as CBT as a tool for behavior change**

Provide knowledge about why and how physical activity patterns affect our mood and how to use tools in cognitive behavioral therapy to change behavior to increase physical activity and reduce sedentary behavior. Discuss the positive effects physical activity has on health and what risks there are with long-term sitting and the importance of taking breaks from long-term sitting. Discuss, for example, the risk of "the active couch potato", i.e. that even for those who exercise, it is important to make breaks in prolonged sitting.

2. **Map the current situation**

Based on the individual's knowledge of the target behavior, to increase physical activity or reduce sedentary behavior, start from the 24-hour illustration in Appendix 5, Alternative Behaviors and discuss how the individual currently moves. Many people need help identifying what they actually do, i.e. when you are physically active, what counts as physical activity and what sedentary behavior means and when you do what.

3. **Motivation and barriers**

a) Examine the individual's motivation for behavior change by asking;

"Why are you interested in increasing your physical activity/reducing your sedentary time?"

b) Investigate the individual's experience of obstacles/difficulties to making the behavior change by asking;

"What have you previously tried (regarding changing your physical activity pattern)?" "
"How did it work?"
"What has stopped you?"
"What have these obstacles cost you?"

Enter the answers in the matrix in field 2, internal obstacles, such as thoughts and feelings (eg I don't like getting sweaty) and field 3, Behaviors that have led the individual away from goals and values (eg I work a lot). The individual's answer to the question about motivation can already be derived here from Values, field 1.

4. Goal and values

Examine the individual's desire for goal setting and connect it to values so that the goals have an emotional meaning.

a) Ask the following questions about values:

"How would you like/wish it was?"

"What is important to you regarding your health?"

"If you think we waved a magic wand and everything was possible, how would you like to take care of yourself in terms of your physical activity pattern?"

b) Ask the following questions about goals:

"What could you do to take steps in the direction of what is important to you?"

"What is the smallest possible step you can take when you walk out of here today?"

Formulate the goals as SMART goals (Specific, measurable, accepted, realistic, time-bound). Start from where the individual is so that the goals are realistic and will really be achievable. Often the individual's goals need to be broken down into several steps.

Enter in the matrix in field 1, Values (eg. My family is important to me and I want to be healthy and energetic so I can play with my children) and field 4, Goals (eg. Weeks 1-2 I stand up at the desk 1 time in the morning, 1 time in the afternoon and 1 time in the evening when I sit and watch TV, for example. When I stand up, I can either do 10 leg bends + 10 toe raises or go for a 2-minute walk. Week 3- 5 I stand up once an hour. From week 6 I stand up once every half hour)

5. VAS-scales

Examine the individual's motivation and confidence in their ability to implement the behavior change by asking;

"How important is it for you to change your activity pattern (physical activity/sedentary, 1-10?"

"What do you think about your chances of passing it, 1-10?"

"How likely is it that you will change your physical activity pattern today, 1-10?"

Use the answers as discussion material and ask follow-up questions.

For example, "How come you don't estimate a lower value?"

Mark on the VAS scales.

6. **Introduce activity diary and activity app**
   Register at least one week between sessions

Homework

Homework 1: Register a week's activity (Activity diary and possibly Activity app)

Homework 2: Continue to explore values and formulate short- and long-term goals (Matrix fields 1 and 4)

Material

- Activity diary (appendix 1)
  The participant fills in at home and presents at the next session. The coach documents and saves in the participant's folder.
- The matrix (appendix 2)
  The participant fills in at home and presents at the next session. The coach documents and saves in the participant's folder.
- VAS scales (Appendix 3). Coach keeps it.

Session 2 – Individual session

1. Write the agenda on the board and ask if the participant is ok with the session being filmed

2. Go through homework

How has the week gone? What was hard? What has gone well? Go through the activity diary and activity app as well as the goals and values filled in fields 1 and 4 of the matrix. Collect and document activity diaries and matrix.

3. Map the current situation

Feedback objective measurements of physical activity or sedentary behavior and link to activity diary and activity app.

4. **Introduce situation analysis**

Make a situation analysis in the session based on a concrete example in the individual's everyday life, to show how thoughts, feelings, behavior and consequences are connected and which alternative behaviors can be imagined in the situation.
Complete the Situation Analysis together based on the individual's example. It can help to start with the behavior and then go to the situation that triggered the behavior (e.g. Behavior: I canceled training with a friend, The situation: I came home from a long and hard day at work, Thoughts: I should exercise but I can't, Feelings: Anxiety because I should, Body reactions: Tired, body aches, Short-term consequence: Relief, relaxed anxiety, relaxed exercise Long-term consequence: Feels even harder next time, more fatigue).

Also discuss possible alternative behaviors that are filled in Field 4 because alternative behaviors constitute goal formulation. Examples of alternative behaviors can be found in Additional file 5.

Homework

Homework 1: Record a week's activity (Activity diary and Activity app).
Homework 2: Situational analysis of at least two situations for next time – thoughts and feelings about training situations/behaviors to reduce sedentary behavior.

Material

- Activity diary (appendix 1)
  The participant fills in at home and presents at the next session. The coach documents and saves in the participant's folder.
- The matrix (appendix 2)
  The participant fills in at home and presents at the next session. The coach documents and saves in the participant's folder.
- Situation analysis (appendix 4)
  The participant fills in at home and presents at the next session. The coach documents and saves in the participant's folder.
- List of alternative behaviors (appendix 5) for the participant to take home.
  Video or audio recording equipment

Session 3 - Group session

1**. Write up the agenda**

2. Current situation
All participants give an update about their situation.

3. Values and goals
The participants share values and goals with each other in the group. Each participant looks in their matrix, fields 1 and 4 and answers the questions:
"Why am I here?"
"What are my goals to get there?"

4. Experience-based practice in Acceptance, to practice accepting troublesome feelings and thoughts
The purpose of the exercise is acceptance/increased willingness of troublesome feelings and thoughts that prevent you from doing things that are important. Keeping the discomfort as far away as possible costs more than accepting it and letting it be there/as it is and acting in a valued direction. Three different experience-based exercises are described below, where the conversation leader chooses one of the exercises to be carried out in the group.

Exercise 1 – "a bag full of things": The facilitator demonstrates the exercise with a volunteer participant. The conversation leader asks the participants if it is ok to share obstacles in the form of thoughts, feelings and behaviors with each other. If not ok, the participants can either choose to refrain from the exercise or create fictitious obstacles. The facilitator instructs the participant holding an empty bag with outstretched arms straight ahead. The interviewer uses a number of books that illustrate troublesome thoughts and feelings that the participant experiences as obstacles to increased physical activity/reduced sedentary time. The interviewer asks the participant for thoughts and feelings that are obstacles and puts book after book in the bag as a metaphor for these. The interviewer asks: How do you feel now? Puts in the next book and asks: How does it feel now? After a number of books, the interviewer asks: What could you do to make it feel less difficult? If the participant suggests that the bag can be thrown away, the conversation leader answers: How has it worked so far? Looks like the bag is coming back? Is there any other way to hold your thoughts and feelings? If the participant suggests that the bag can be held close to the body, the interviewer answers: Do you want to try? How does it feel now? What has this exercise given you? Often the participants answer that it feels easier to keep troublesome thoughts and feelings close to the body, and then the conversation leader confirms this: Yes exactly, keeping our troublesome thoughts and feelings close instead of fighting against them makes them affect us less. The participants then pair up and do the exercise on each other. One person puts in books and the other holds the bag. Each individual looks in their matrix, fields 2 and 3, for examples of hindering thoughts, feelings, and behaviors.

Exercise 2 – "ACT in a Nutshell": The interviewer demonstrates the exercise with a volunteer participant. The conversation leader asks the participants if it is ok to share obstacles in the form of thoughts, feelings and behaviors with each other. If not ok, the participants can either choose to refrain from the exercise or create fictitious obstacles.

The interviewer instructs the participant who holds up a book or sheet of paper/binder in front of the participant. Imagine that this book/binder represents troublesome thoughts, feelings and memories that you have struggled with for years to change your physical activity pattern. The interviewer gives the book/binder to the participant. The interviewer says “Hold it as tight as you can and hold it in front of your face, so you can't see me. Hold it so close to your face that it next touches the tip of your nose”. The participant cannot now see the conversation leader or others in the room. The conversation leader asks the participant how it feels to have a conversation with the cardboard in front of their face. The conversation leader asks if the participant can feel contact with the conversation leader and determine what facial expression he has and if the conversation leader moved, would the participant be able to note this? The interviewer also asks the participant to describe what the room looks like, with the book/binder still in front of the face. The participant will find it difficult to do all this, which is why the interviewer says "so when you are absorbed in your thoughts, feelings and painful memories, you miss a lot of things and lose touch with what is important to you. For example, if I asked you to start doing push-ups and you still held the cardboard in front of your eyes, it would be difficult to perform the exercise, and if you were to physically succeed in doing push-ups, all your strength and energy would still be spent holding the book/binder in place . Also, if you often struggle to get rid of these unpleasant experiences, which are thoughts, feelings and memories, not only can it be difficult to stay focused on what's important, but the whole thing of keeping the unpleasant away becomes a struggle which I intended to show you through the metaphor”. The interviewer asks the participant to keep the book/binder as far away from them as possible. The interviewer holds the other side of the book/binder.
The interviewer asks the participant if he likes having this discomfort in front of him? The interviewer asks the participant to try to suppress the troublesome thoughts, feelings and memories. The interviewer asks the participant to push away from him as hard as he can, whereby the interviewer pushes back in the opposite direction. The interviewer asks the participant how it feels in the arms and body. The interviewer says that "we've only been at it for a short while now but what would it be like to fight this fight for years, what would it lead to in the long run?" The interviewer asks the participant to stop the exercise and ask what experiences he got from the exercise.

Exercise 3 – “your hands are the problem”: The conversation leader does the exercise on himself and asks all participants to do it together with him. The interviewer says “Place your hands in your lap with the palms facing up. If your hands were to symbolize your thoughts, feelings and painful memories (think: the matrix square 2 and 3) and you now slowly bring your hands up to your face. Now imagine that all the things you have struggled with and perhaps failed with in relation to your physical activity pattern will soon occupy your entire field of vision. Let the hands touch the nose and cover the whole face". The conversation leader asks how it feels for the participants in the group to be overwhelmed by this discomfort. The interviewer then asks the participants to slowly put their hands in their laps again and observe the difference between the experiences. Willingness to have these troublesome thoughts, feelings and memories is an important part of being able to let the discomfort "lie in your lap" and direct your attention to what is important. The metaphor aims to give the participants their own experiential experience of what acceptance and willingness can be for her or him.

5. Summary

The facilitator asks all participants to share what they took away from the session.

Collect, document and save activity diaries, situational analyzes and matrices.

All participants take accelerometers home with them, which they wear for a week and then send back in the enclosed envelope. A reminder is added to the calendar together with the participant during the session.

Hemuppgifter

Hemuppgift 1: Registrera en veckas aktivitet (Aktivitetsdagbok och eventuellt Aktivitetsapp).

Hemuppgift 2: Situationsanalys på minst två situationer till nästa gång – tankar och känslor kring träningssituationer/beteenden för att minska stillasittande.

Material

- Aktivitetsdagbok (bilaga 1)
  Deltagaren fyller i hemma och visar upp vid nästa session. Samtalsledaren dokumenterar och sparar i deltagarens mapp
- Fyrfältaren (bilaga 2)
  Deltagaren fyller i hemma och visar upp vid nästa session. Samtalsledaren dokumenterar och sparar i deltagarens mapp
- Situationsanalys (bilaga 4)
  Deltagaren fyller i hemma och visar upp vid nästa session. Samtalsledaren dokumenterar och sparar i deltagarens mapp

Homework

Homework 1: Register a week's activity (Activity diary and possibly Activity app).

Homework 2: Situational analysis of at least two situations for next time – thoughts and feelings about training situations/behaviors to reduce sedentary behavior.

Material

- Activity diary (appendix 1)
  The participant fills in at home and presents at the next session. The coach documents and saves in the participant's folder
- The matrix (appendix 2)
  The participant fills in at home and presents at the next session. The coach documents and saves in the participant's folder
- Situation analysis (appendix 4)
  The participant fills in at home and presents at the next session. The coach documents and saves in the participant's folder

Session 4 – Individual session

1. Write up the agenda

2. Map the current situation

Feedback objective measurements of physical activity or sedentary behavior and link to activity diary and activity app.

3. Go through homework

How has the week gone? What was hard? What has gone well? Go through situation analyzes and activity logs.

Collect and photograph activity diaries, matrix and situation analyses

4. Evaluate goal achievement

Get in touch with values and, if necessary, revise goals (when the change in physical activity or sedentary life is to be done gradually and successively increased, it is appropriate to increase the goals here). Examine which obstacles the individual has overcome, and what he needs to overcome more obstacles. Discuss alternative behaviors with the participant to the problematic situations in the situation analyses. Alternative behaviors are entered as goal formulation. Feel free to get in touch with the values to find alternative behaviors that link values to goals.

Enter in mateix, field 4.

5. Create triggers for alternative behavior

Investigate and create triggers for alternative behavior (e.g. putting on exercise clothes in the morning, standing desk in a standing position, etc.)

6. Introduce summary and sustainment plan

Summarize what you worked on and how the individual can continue with the behavioral change.

The participant begins filling out the Summary and Retention Plan in the session. The individual then continues filling in the plan at home.

Homework

Homework 1: Record a week's activity (Activity diary and Activity app).

Homework 2: Situational analysis of at least two situations for next time – thoughts and feelings about training situations/behaviors to reduce sedentary behavior

Homework 3: Sustainment plan and setback management.

Material

– Activity diary (appendix 1)
The participant fills in at home and presents at the next session. The coach documents and saves in the participant's folder
– The matrix (appendix 2)
The participant fills in at home and presents at the next session. The coach documents and saves in the participant's folder
– Situation analysis (appendix 4)
The participant fills in at home and presents at the next session. The coach documents and saves in the participant's folder
– Alternative behaviors (appendix 5)
The participant fills in at home and presents at the next session. The coach documents and saves in the participant's folder
– Summary and sustainability plan (Appendix 6)
The participant fills in at home and presents at the next session. The coach documents and saves in the participant's folder

Session 5 - Group session

1. Write up the agenda

2. Maintenance plan

Participants share maintenance plans and setback management with each other by answering the questions:

"How do I maintain my physical activity pattern?"

"What do I do when I have a setback?"

3. Summary

All participants tell us what they took away from the conversation support during the past weeks.

Collect, document and save activity diaries, matrices, situational analyzes and maintenance plans

Material

– Activity diary (appendix 1)
The coach documents and saves in the participant's folder
– The matrix (appendix 2)
The coach documents and saves in the participant's folder
– Situation analysis (appendix 4)
The coach documents and saves in the participant's folder
– Continuation plan (Appendix 7)
The coach documents and saves in the participant's folder
